# Network analysis of inflammatory responses to sepsis by neutrophils and peripheral blood mononuclear cells

**DOI:** 10.1371/journal.pone.0201674

**Published:** 2018-08-07

**Authors:** Rasoul Godini, Hossein Fallahi, Esmaeil Ebrahimie

**Affiliations:** 1 Department of Biology, School of Sciences, Razi University, Baq-e-Abrisham, Kermanshah, Iran; 2 Adelaide Medical School, The University of Adelaide, Adelaide, Australia; 3 Institute of Biotechnology, Shiraz University, Shiraz, Iran; 4 School of Information Technology and Mathematical Sciences, Division of Information Technology, Engineering and the Environment, The University of South Australia, Adelaide, South Australia, Australia; 5 School of Biological Sciences, Faculty of Science and Engineering, Flinders University, Adelaide, South Australia, Australia; Auburn University College of Veterinary Medicine, UNITED STATES

## Abstract

Sepsis is a life-threatening syndrome causing thousands of deaths yearly worldwide. Sepsis is a result of infection and could lead to systemic inflammatory responses and organ failures. Additionally, blood cells, as the main cells in the immune systems, could be also affected by sepsis. Here, we have used different network analysis approaches, including Weighted Gene Co-expression Network Analysis (WGCNA), Protein-Protein Interaction (PPI), and gene regulatory network, to dissect system-level response to sepsis by the main white blood cells. Gene expression profiles of Neutrophils (NTs), Dendritic Cells (DCs), and Peripheral Blood Mononuclear Cells (PBMCs) that were exposed to septic plasma were obtained and analyzed using bioinformatics approaches. Individual gene expression matrices and the list of differentially expressed genes (DEGs) were prepared and used to construct several networks. Consequently, key regulatory modules and hub genes were detected through network analysis and annotated through ontology analysis extracted from DAVID database. Our results showed that septic plasma affected the regulatory networks in NTs, PBMCs more than the network in DCs. Gene ontology of DEGs revealed that signal transduction and immune cells responses are the most important biological processes affected by sepsis. On the other hand, network analysis detected modules and hub genes in each cell types. It was found that pathways involved in immune cells, signal transduction, and apoptotic processes are among the most affected pathways in the responses to sepsis. Altogether, we have found several hub genes including ADORA3, CD83 CDKN1A, FFAR2, GNAQ, IL1B, LTB, MAPK14, SAMD9L, SOCS1, and STAT1, which might specifically respond to sepsis infection. In conclusion, our results uncovered the system-level responses of the main white blood cells to sepsis and identified several hub genes with potential applications for therapeutic and diagnostic purposes.

## Introduction

Sepsis, a life-threatening condition, occurs when the immune reaction against an infection diverted toward the healthy cells in the body. Serious conditions including organ dysfunction and hypotension refractory have also been observed in “severe sepsis” and “septic shock”, respectively [1]. More than 750,000 cases of severe sepsis (with mortality rates of 30 percent) have been reported in the US [2]. Additionally, sepsis-related costs estimated to be more than 20 billion dollars in 2011 in the US [3]. Therefore, many studies have been conducted to address different aspects of this disease. At the molecular level, a link between “cytokine storm” and sepsis has been established, which is defined as the abnormal increase in the levels of different cytokines, including tumor necrosis factor (TNF) and interleukin-1 (IL-1) [4]. Perturbations in the cardiovascular system, acute kidney injuries, encephalopathy, and immobility are among organ-level responses to sepsis. Sepsis has also associated with the alterations in many cellular processes including inflammatory signaling, metabolic pathways, and resolution pathways [5].

Any immune response involves different cell types and molecules. For instances, it is known that neutrophils, dendritic cells, and peripheral blood mononuclear cells are involved in responses to sepsis. Neutrophils (NTs) and monocytes are among the most important WBCs, which together with T cells, B cells and dendritic cells constitute the cellular components of the immune systems [6–8]. NTs are known for their phagocytosis activities against pathogens and particles. These cells are also establishing a communication network in triggering inflammation responses through induction of platelet formation and production of inflammatory cytokines by macrophages [9]. NTs are involved in many important phenomena including chronic inflammatory disease [10], neurodegenerative responses [11], and wound healing [12]. On the other hand, monocytes are the main precursors of the immune cells such as tissue macrophages. Monocytes, dendritic cells (DCs) and mononuclear phagocytes are the three subgroups of the mononuclear phagocytes. Presentation of antigens, phagocytosis, and immunomodulation are the three main functions of blood mononuclear phagocytes, which indicate their importance for both innate and acquired immunity [13]. By analyzing the transcription dynamics in these cells, following exposure to sepsis, one might be able to establish the underlying molecular processes.

However, analyzing the changes in gene expression in the septic patients might not be useful because of the complexity of responses and involvement of multidimensional networks of molecules and cells. Therefore, assessing the responses to sepsis by individual types of cells might be more informative. On the other hand, as sepsis is a system-wide phenomenon, it needs a system-level approach to identify the involved pathways and regulatory elements. Network analysis is one of such approaches that produce valuable information and identifies underlying processes by looking at the whole system, using either transcription or proteomics data [14]. To abstract the biological significance of different parts of the network, the nodes could be grouped to modules, which are related to specific functions. Using network analysis, not only modules of activities, but highly connected genes could be also identified, which are known as “hub genes” [15].

Here, we have investigated the mechanism by which the WBCs respond to the septic plasma, obtained from septic patients, as a source of inflammatory chemicals and extract of microbes. We have dissected and compared the molecular responses by NTs, DCs and Peripheral Blood Mononuclear Cells (PBMCs) in isolated media, using Weighted Gene Co-expression Network Analysis (WGCNA) [16], PPIs [17], and GRNs [18] methods. These methods can detect systems-level processes and hub genes as the most important factors in this event. Our analysis showed that immune cells, signal transduction, and apoptotic processes are among the first line of responses to sepsis. We also showed that NTs and PBMCs response more similar to sepsis compared to that of DCs.

## Material and methods

### Data preprocessing and expression analysis

The matrices containing the expression values, provided by the authors on the GEO datasets, were downloaded. The matrices were then normalized, log2 transformed and filtered using PALO (keeping only those genes with a *p-value* ≤ 0.01 in at least one sample). The final gene expression matrices for DCs, NTs, and PBMCs contained 23,589, 21,236, and 25,728 genes, respectively. For WGCNA, we first calculated the coefficient of variation and then selected the most variable probes (the top 0.70 percentile). By removing duplicate symbols, our final matrices for WGCNA contained 12,500, 11,217, and 13,459 probes in DCs, NTs, and PBMCs, respectively. To construct other networks, differentially expressed genes (DEGs) were detected using limma package, embedded in GEO2R tool of NCBI (http://www.ncbi.nlm.nih.gov/geo/geo2r/). Each sample was individually assessed for possible outliers, using the method supplied in WGCNA package. Then, using average methods, clustering was conducted to detect dissimilarities between samples. The lists were further filtered to remove the genes with a *p-value* ≤ 0.05. Expression analyses were conducted on the Log2 transformed values, therefore we have selected ±0.6 Log2 of fold changes (FC) as the threshold for DEGs (representing 1.5-fold differences at the fold change level). The DEGs lists were manually checked to remove possible none unique genes.

### WGCNA analysis

For each cell type, the network was constructed after calculation of the Pearson correlations between pairs of genes across all samples [19]. To construct a scale-free topology network (unsigned network), adjacency matrix was calculated by applying soft thresholding power of 8 to the correlation data. This threshold was selected taking into account both the scale-free topology fit index 0.9 (R^2^ = 0.9) and the number of samples (30 samples in the current study). Then, the adjacency matrix transformed into topological overlap matrix and finally, the scale-free topology network was constructed from its dissimilarity values. Gene modules in the resulting networks were detected by the dynamic hybrid tree cut algorithm and those with an eigengenes correlation of 0.75 and above were merged. Modules eigengenes can be considered as the principal component of modules. Afterward, module eigengenes correlations with traits, including disease status, were calculated to identify those modules related to the disease. Highly correlated modules to the status of disease were further analyzed to find gene-module membership and gene significance correlation. Genes with high membership and correlation scores were used to construct another set of networks. The expression patterns were also investigated for all gene members of the modules and the hub genes correlate with the disease status.

### PPI network analysis

PPI network was constructed using information obtained for the DEGs lists following submission to the STRING database [20]. We have only included those interactions supported by experiments. To extract valid interactions, the confidence level was set at 0.40, which was used as the minimum interaction score (this will give a medium confidence, please refer to the STRING database manual). The resultant network was visualized and analyzed in Cytoscape 3.4.0 [21] and Gephi v 0.9.1 [22]. Several modules in the network were identified by overlapping neighborhood expansion algorithm through ClusterONE 1.0 plugin [23]. Hub genes were detected based on centrality analysis factors including degree and betweenness [24]. In order to do this, we first sort DEGs in the PPI networks based on degree and betweenness and selected the top 10 percent of all nodes. Those genes that harbor both higher degree of connectivity and betweenness were considered as hubs and used for further analysis.

### Gene regulatory network analysis

To construct a gene regulatory network, we have checked the DEGs lists for the presence of TFs and the interactions between genes and TFs were investigated. The lists of DEGs were submitted to Tfcheckpoint [25] and ChEA database [26] to detect both the TFs in the lists of DEGs and those TFs that are known to regulate the genes in the DEGs, respectively. For detection of TFs, the library of Tfcheckpoint database was downloaded, which contains manually curated TFs with experimental evidence. Altogether, we have obtained a list of 882 TFs with DNA-binding features. For finding those TFs controlling the genes on the DEGs lists, these lists were submitted to ChEA database. This database contains data on protein-DNA interactions provided by ChIP-X experiments. We have only selected those TFs that show a *p-value* below 0.1 and being differentially expressed. Networks were constructed based on pairs of TF-target interactions and centrality analysis was performed on the network afterward.

### Gene ontology analysis

To identify involvement of possible functions and processes, gene ontology was performed using DAVID database [27]. We only considered information regarding gene ontology (GO), biological process (BP) and Kyoto Encyclopedia of Genes and Genomes (KEGG) in our analysis. Top 10 terms with highest numbers of members and a *p-value* below 0.1 were selected for the analysis.

## Results

### Gene expression analysis and annotation

**Fig 1:**
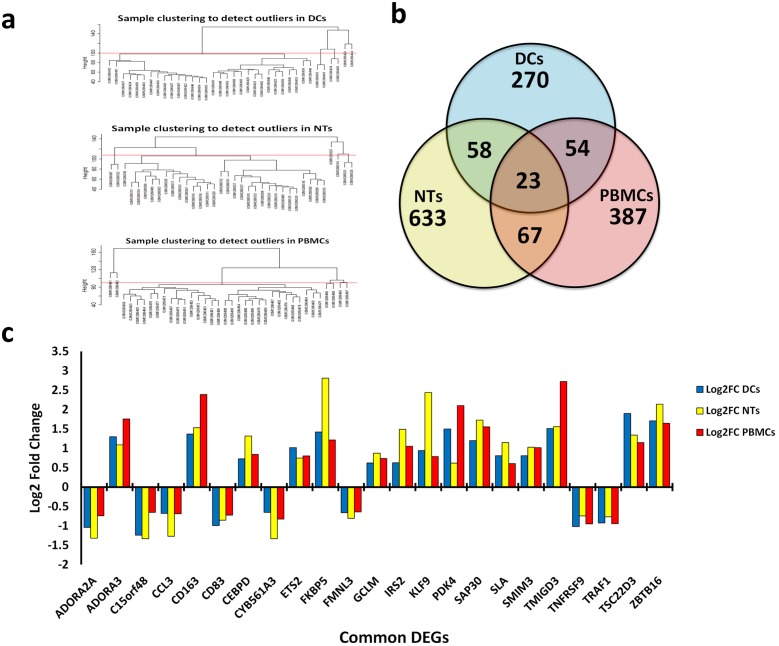
DEGs detection. a) Clustering of samples to detect outliers in each cell line. b) Venn diagram of the common DEGs among the cell types. c) Bar chart of the fold change of expression among 23 common DEGs among all cells. Abbreviations: DEGs: Differentially Expressed Genes; DCs: Dendritic Cells; NTs: Neutrophils; PBMCs: Peripheral Blood Mononuclear Cells.

**Fig 2:**
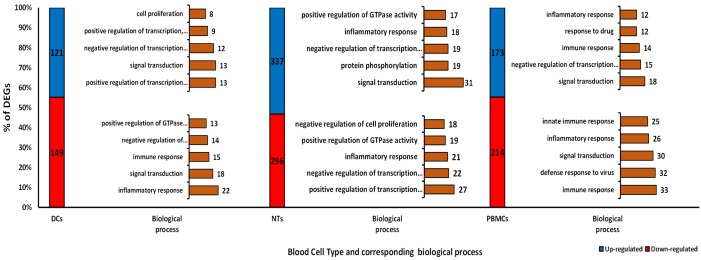
The count and percentage of DEGs in each cell types. Top 5 biological processes of both up-and down-regulated genes related to each type of cells are presented in right of the bars. Abbreviations: DEGs: Differentially Expressed Genes; DCs: Dendritic Cells; NTs: Neutrophils; PBMCs: Peripheral Blood Mononuclear Cells.

**Fig 3:**
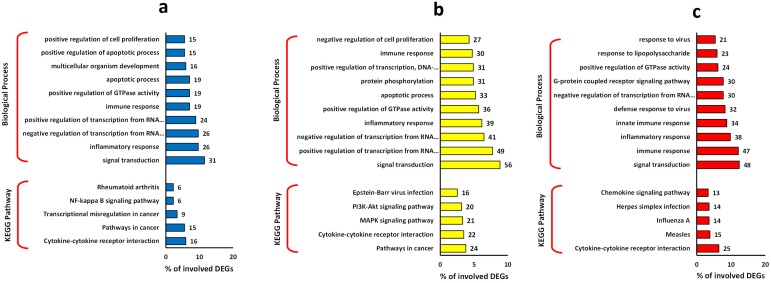
Gene ontology of the DEGs. Top 10 biological process and top 5 KEGG pathway are presented for a) DCs, b) NTs, and c) PBMCs. Abbreviations: DEGs: Differentially Expressed Genes; DCs: Dendritic Cells; NTs: Neutrophils; PBMCs: Peripheral Blood Mononuclear Cells.

In this study, we have analyzed the impacts of septic plasma on cultured NTs, DCs, and PBMC cells. Treatment of NT cells with septic plasma resulted in significant changes in the expression of 633 genes (whereby called DEGs), much fewer genes, 387 and 270 DEGs were affected by sepsis in PBMCs and DCs, respectively (Fig 1a). Additionally, we found 67 common DEGs between NTs and PBMCs, 58 for DCs and NTs and 54 between DCs and PBMCs (Fig 1b). Interestingly, we found only 23 common DEGs among all type of cells, where 8 genes were down-regulated and 15 genes shown up-regulation (Fig 1c). Gene ontology of these 23 common DEGs showed that apoptotic process (TRAF1, TNFRSF9, ADORA2A, ZBTB16), negative regulation of transcription (SAP30, TSC22D3, ETS2, ZBTB16), and inflammatory response (TNFRSF9, CCL3, ADORA2A) are the main biological processes affected by sepsis treatment. Most DEGs were down-regulated in DCs and PBMCs, but opposite trend was observed for the NTs samples (Fig 2). Gene ontology of DEGs clearly revealed that signal transduction and inflammatory response are the most important biological processes affected by sepsis in all these cell types (Fig 3). The apoptotic process was also prominent biological process affected in response to sepsis, which was followed by transcription regulation process. Furthermore, KEGG pathway analysis revealed the cytokine-cytokine receptor interaction as the common process responding to sepsis exposure. While, it is hard to speculate if these pathways are upregulated or downregulated, based on the number of the involved DEGs, it appears that down-regulation of immune and inflammatory response has occurred.

### PPI analysis

**Fig 4:**
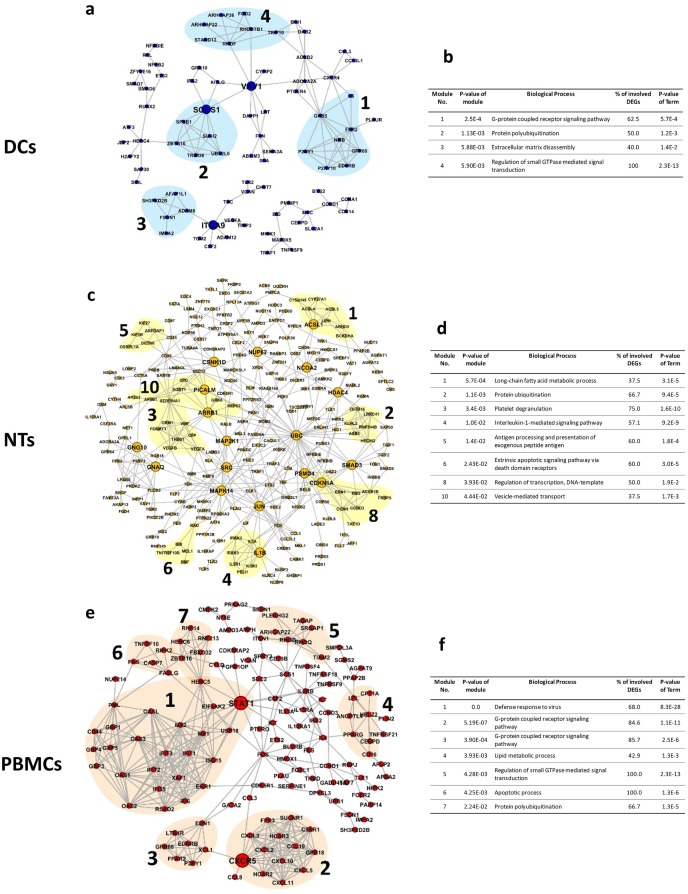
PPI networks and module annotations. PPI networks of the cell types are presented. Numbers and colorful shades indicate the modules detected by overlapping neighborhood expansion. Tables show the annotation and *p-values* of the modules corresponding to the networks. a, b) Network and counterpart table of annotations for DCs, c, d) Network and counterpart table of annotations for NTs, and e, f) Network and counterpart table of annotations for PBMCs. Abbreviations: DEGs: Differentially Expressed Genes; DCs: Dendritic Cells; NTs: Neutrophils; PBMCs: Peripheral Blood Mononuclear Cells.

We have constructed several PPI networks using DEGs and protein interactions information obtained from STRING database. Network analysis revealed that NTs network contained 307 nodes and 550 edges (Fig 4). DCs network had the smallest size, with 108 nodes and 148 edges. The networks were analyzed to detect functional modules and identify possible underlying processes. Altogether, 4, 10 and 7 modules were detected for DCs, NTs, and PBMCs networks, respectively; using overlapping neighborhood expansion algorithm and a *p-value*<0.05. These modules are involved in protein ubiquitination and G-protein coupled receptor signaling pathway. Expectedly, processes related to phagocytosis, including vesicle-mediated transport and long-chain fatty acid metabolic process, were detected in the NTs network. Next, we have identified the hub genes by choosing those nodes with the highest degree of connections (top 10%) and betweenness. Collectively, three hub nodes (ITGA9, SOCS1, VAV1) were found in the DCs network. While, NTs network contained 18 hubs, including IL1B, JUN, SMAD3, and UBC. Only two hubs, namely CXCR5 and STAT1, were detected for PBMCs network. Surprisingly, only a few of these hubs belonged to the functional modules, indicating the importance of identifying hub genes rather than analyzing functional modules alone.

### Transcription factors analysis

**Fig 5:**
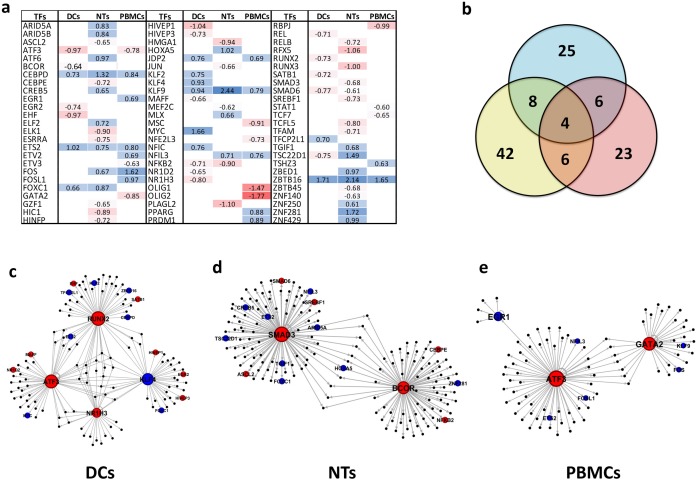
TFs and GRN analysis. a) Detected TFs in all cell types and their expression status. Blank spaces mean no expression alteration was observed for the TFs. b) Venn diagram of common TFs among cell types. c) GRN of DCs, d) GRN of NTs, and f) GRN of PBMCs. Blue colors mean up-regulation and red mean down-regulation. Abbreviations: TFs: Transcription Factors; GRN: Gene Regulatory Networks; DCs: Dendritic Cells; NTs: Neutrophils; PBMCs: Peripheral Blood Mononuclear Cells.

We have detected 25, 42, and 23 differentially expressed TFs after treatment of DCs, NTs, and PBMCs with septic plasma. Fig 5a shows the dispersion and expression status of these TFs in different cells. DCs and NTs contained more down-regulated TFs, while the majority of TFs in PBMCs were up-regulated. Interestingly, all common TFs showed similar expression pattern, with only one exception TSC22D1. Our results indicate that CEBPD, ETS2, KLF9, and ZBTB16 were common up-regulated TFs among all cells (Fig 5b). In the GRNs constructed for the sepsis-treated DCs, the TFs that are involved in the gene regulation were identified. They include ATF3, KLF4, NR1H3, and RUNX2. Similarly, BCOR and SMAD2 were found in the NTs network. It appears that the response to sepsis in the PBMCs cells is controlled through ATF3, EGR1, and GATA2 activity. Notably, the identified TFs were down-regulated in response to sepsis, except EGR1 and KLF4 (Fig 5c, 5d and 5e). Further, we have observed an inverse expression trend between many of these TFs and their primary targets. For instance, all target of ATF3 in PBMC treated with sepsis plasma were up-regulated.

### WGCNA analysis

**Fig 6:**
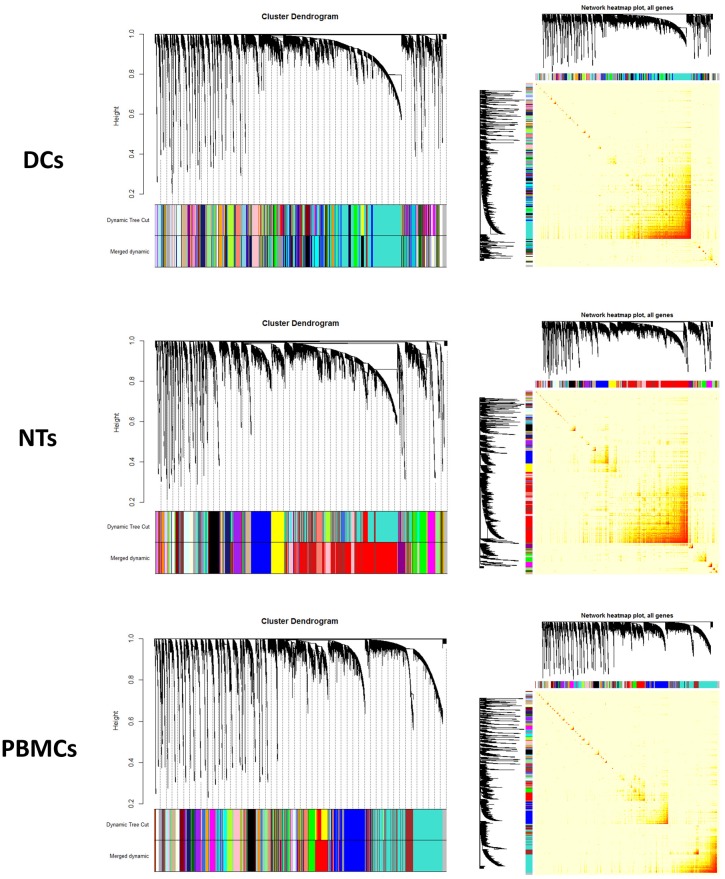
Module detection and network heat map plot construction using WGCNA. a, b) cluster dendrogram and network heat map plot of all genes of DCs. c, d) cluster dendrogram and network heat map plot of all genes of NTs. e, f) cluster dendrogram and network heat map plot of all genes of PBMCs. Abbreviations: DCs: Dendritic Cells; NTs: Neutrophils; PBMCs: Peripheral Blood Mononuclear Cells.

**Fig 7:**
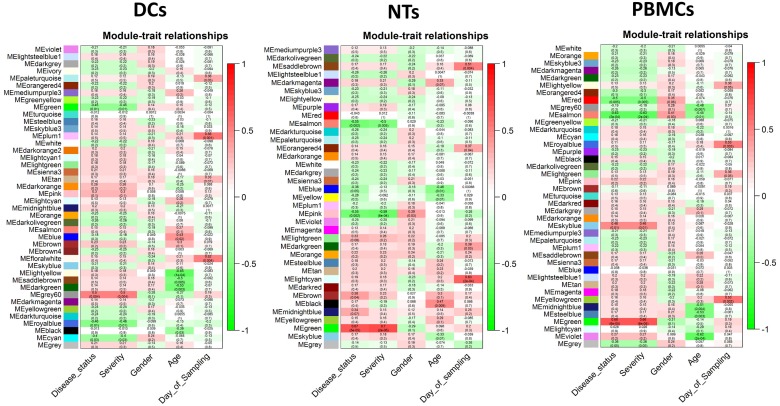
Heat maps of module eigengenes relationships with traits. a) heat map for the traits of dendritic cells, b) heat map for the traits of neutrophils, and c) heat map for the traits of peripheral blood mononuclear cells.

WGCNA is constructed based on pairwise gene expression correlations extracted from the matrix of gene expression values (Fig 6). In the resulting network, the modules were detected by clustering method based on the dissimilarity scores. The modules with similarity score higher than 75 percent were merged and reported as one larger module. Finally, 42 modules were identified in the DCs, 37 in the NTs, and 41 in the PBMCs networks in response to septic plasma (Fig 7). A strong correlation was observed between some of these modules and the disease. We have only further analyzed those modules that showed an R^2^ ± 0.3 (with a *p-value* ≤ 0.05) correlation between gene/module membership and the diseases. As a result, three modules were detected that were highly correlated or anti-correlated with the disease status (Fig 7). Then we have assessed that if the genes in these modules show differential expression. The results clearly showed that at least 10 percent of the genes in each module are among DEGs. Expectedly, the expression pattern of the DEGs followed similar pattern observed for the modules/trait correlation. For instance, up-regulated genes are dominant in the module marked as “grey60” in the treated DCs, which is correlated with disease status (correlation = 0.5 and *p-value* = 0.004). We have also investigated if the modules related to sepsis induction are also related to other traits such as age, gender, ethnic background, etc. We found only one significant correlation between age and “salmon module” in PBMCs, the rest did not show any significant correlation with other traits.

**Fig 8:**
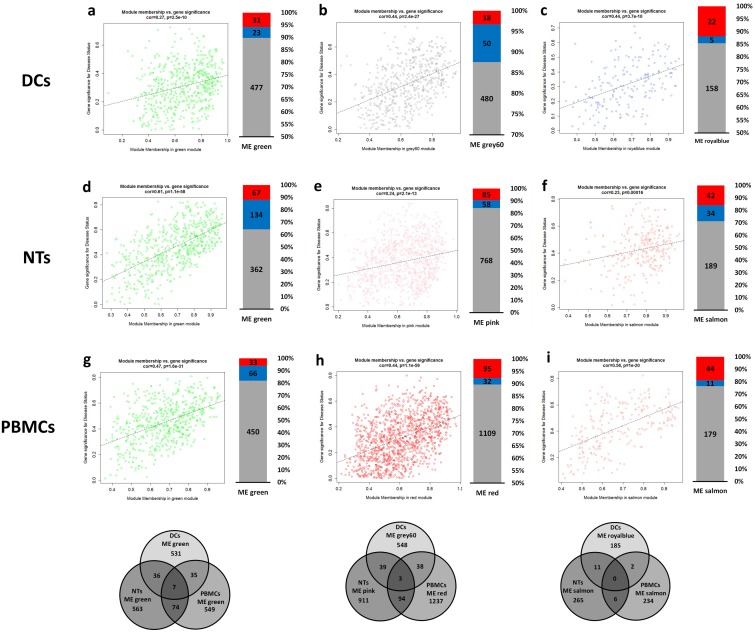
Correlation of module memberships versus gene significance for disease status. Scatterplot graphs show the correlation of module memberships of the genes in each module versus significance of the genes for the disease status. Additionally, expression status of the module members was considered and shown in the form of single bar graphs on the right of counterpart scatterplot graphs. Red and blue colors mean down- and up-regulation. Finally, common genes among the modules were presented in Venn diagram graphs below the related scatterplots of all cell types. No common genes were detected among modules of a cell type. a, b, c) modules of DCs. d, e, f) modules of NTs. g, h, i) modules of PBMCs. Abbreviations: DEGs: Differentially Expressed Genes; DCs: Dendritic Cells; NTs: Neutrophils; PBMCs: Peripheral Blood Mononuclear Cells.

Next, we asked if there are any common genes among identified modules, either within or between different cell types. No common genes were detected among modules by comparing any individual cell types. However, we have identified several identical genes in the modules of different cell types. The highest number of common genes were found for “green modules”, having 7 common genes among all cell types. On the other hand, pairwise comparisons resulted in detection of 36 common genes between DCs and NTs, 74 between NTs and PBMCs, 35 between DCs and PBMCs. Besides, module “grey60” of DCs had 39 common genes with module “pink” of NTs and 38 genes with module “red” of PBMCs. Altogether, three identical genes were found in all of these modules. Lowest number of common genes was detected for the module “royal blue” and “salmon” (Fig 8).

In the next step, we have studied the gene ontology of these key modules. Interestingly, signal transduction, transcription, immune response, and apoptotic processes were the most prominent processes in the detected modules. KEGG annotation also showed that processes related to immune response (including chemokine signaling, cytokine-cytokine receptor interaction) and metabolic pathways are dispersed among modules (Fig 8). Notably, similar processes were present in different modules. In DCs response network, transcription, apoptotic and regulation of apoptotic were associated with both modules “green” and “grey60”, in spite of the fact that they have no common genes. In the NTs’, processes linked to inflammatory response could be detected in all modules. Despite inconsistency in the modules annotation, we found that inflammatory response encompasses a substantial number of genes. These results show that different groups of co-expressed genes are involved in different aspects of inflammatory response because they are grouped in entirely distinct modules with no obvious gene overlaps.

**Fig 9:**
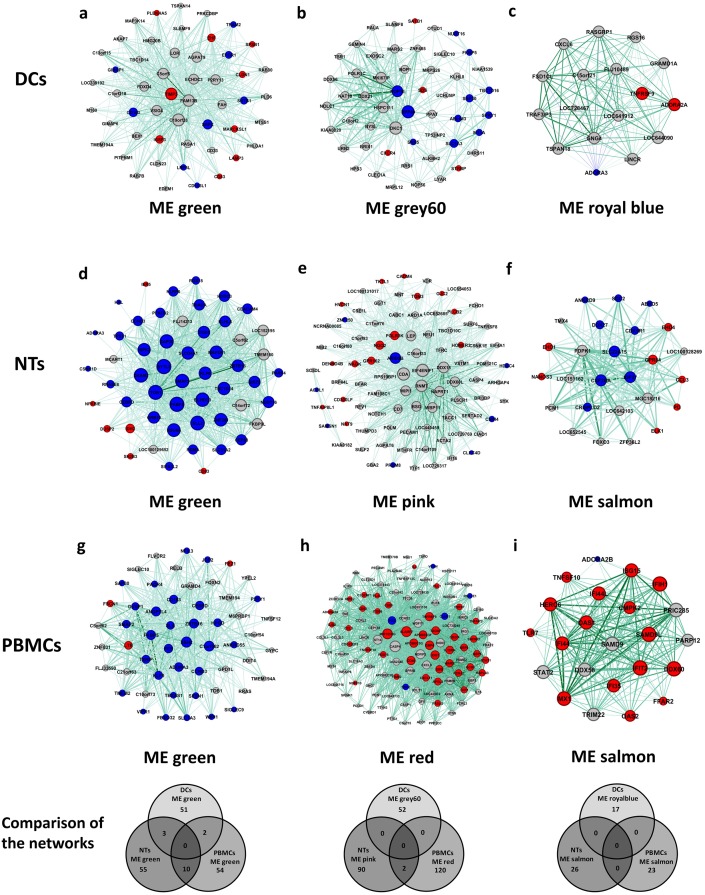
Networks extracted from highly correlated modules to disease. Using top 10 percent hub genes from the modules we constructed networks of each module and detected DEGs among them. Edges were decreased to leave only about top 25 percent weighted edges. Ticker edge means higher weight. Red and blue colors of nodes show either down- and up-regulated genes, respectively. Venn diagrams at below the networks show common genes in the networks from different cells. No common genes were detected among networks of a cell type. a, b, c) networks of DCs. d, e, f) networks of NTs. g, h, i) networks of PBMCs. Abbreviations: DEGs: Differentially Expressed Genes; DCs: Dendritic Cells; NTs: Neutrophils; PBMCs: Peripheral Blood Mononuclear Cells.

To further investigate the role of key regulatory elements, hub genes were used to construct new subnetworks for each cell types (Fig 9). In addition to being hub genes, the selected genes had a high modular membership score, indicating their importance at the system-level response to sepsis. As the networks were very condensed due to the high number of edges, we have used edge weight filter to select only the top 25 percent of nodes. To analyze the networks, we have employed degree centrality factors to detect most connected genes. In some cases, such as “royal blue” in the DCs network or “salmon” in the PBMCs network, almost all genes had similar scores for the degree. There were no identical genes among networks from the same cell types, but regarding networks from different cells, we have only detected a few identical genes in the “green” subnetwork (Fig 9). There were also two common genes in the “pink” and “red” subnetwork of NTs and PBMCs, respectively. We have also checked if the hub genes binged differentially expressed in the original dataset. A correlation between expression status of DEGs of the subnetworks and the majority of DEGs in the modules were observed. In most cases, the DEGs were also hub genes of the networks. The lowest counts of the DEGs were observed in the DCs subnetworks. Here, only 33% and 29% of the genes in the “green” and “grey60” subnetworks were differentially expressed. In comparison, the subnetworks of NTs and PBMCs contained more DEGs, such that “green” subnetworks of NTs and PBMCs contained 85% and 61% DEGs, respectively.

## Discussion

In this work, we have studied the system-level responses of white blood cells to sepsis, using network-based approaches. It is well known that sepsis is a critical and life-threatening syndrome, which is associated with dysregulation of systemic inflammatory response [1]. Interestingly, due to the nature of experimental design, we were able to study individual cell type responses to sepsis, rather than systemic immune system reactions. All three main types of WBCs including DCs, NTs, and PBMCs were exposed to sepsis and their transcriptomes have been studied by Khaenam *et al*. [16]. The original study investigated the molecular signatures to be used as markers for sepsis severity and functions related to the illness. Therefore, that work did not address many questions regarding molecular and system-level responses to sepsis by these three cell types. Hence in the current study, not only DEGs but also co-expressed genes were analyzed through rigorous network analysis to detect gene groups and hub genes performing important roles in individual WBCs responding to septic stimulation.

Our analysis revealed that the total number of DEGs in NTs was higher than the other two cells, showing critical roles of NTs in innate responses and its severe responses to sepsis infection [28]. Gene ontology of the DEGs revealed that signal transduction encompassed the highest number of genes in all cell types, supporting the involvement of signal molecules in the recruitment of immune systems components upon exposure to sepsis. However, it is unknown if the same molecules are involved in the elucidation of a response by all types of cells or different pathways are involved. Previously, it has been shown that toll-like receptors (TLRs) are among the main signaling pathways in WBCs including DCs [29] and NTs [30]. The involvement of TLRs in sepsis has been discovered and discussed previously [31]. Deregulation of the genes related to apoptosis is another prominent event in the sepsis-treated cells that was uncovered by this study. Apoptotic events have been observed in the DCs and NTs cells collected from the blood of septic patients [32, 33]. Transcription alteration was the other important process found to be involved in responses to sepsis, which is in line with previous reports [34]. Interestingly, we have detected similar expression patterns for some genes. This is an indication of the involvement of overlapping processes and pathways between these three cell types.

Similarly, annotation of modules in the networks highlighted the roles of inflammatory response as the main process affected by sepsis in these cells. These findings are not surprising, knowing that NTs and PBMCs make a substantial part of the white blood cells and DCs are central cells contributing to the overall immune system responses [10, 13]. Previous studies showed that DCs affected by sepsis decrease the synthesis of inflammatory factors [35]. Our results showed that DCs’ inflammatory response genes are down-regulated. In two other cells, a mixed pattern of both up- and down-regulation was observed for the genes involved in immune response. Studying the gene-expression profile of NTs has also revealed down-regulation of genes related to inflammatory responses [36]. In PBMCs cells, unexpectedly, we have found that immune response genes undergone both down- and up-regulation. Results obtained from GRNs were also consistent with these findings. Our results unraveled that sepsis dysregulates the network of genes involved in inflammatory response rather than affecting single genes.

Interestingly, the results of network analysis confirmed the overall results obtained from DEGs annotation. Immune cells response, signal transduction and transcription regulation were the main processes in the networks constructed for all three classes of WBCs. However, the results have also identified cell-specific responses to sepsis. Detected sub-networks in the PPI networks, was particularly related to the main aforementioned processes. For instance, protein ubiquitination, associated with apoptosis [37], is observed in all cells in addition to Interleukin-1-mediated signaling pathway and response to virus infection. We could not detect any modules in the network with GO similar to that obtained from DEGs annotations, implying the need for analyzing gene expression at the system-level rather than looking at individual differentially expressed genes. Accordingly, many of the previously described processes, such as immune response, could not be linked to the detected modules. This was despite the fact that their corresponding genes were used to construct the networks. Similar findings obtained from the WGCNA. These types of networks could identify the correlations between different genes solely based on the expression values. Collectively, annotation of the modules in these networks highlighted the role of the same processes in sepsis response, similar to that observed in DEGs and PPI analysis. This information clearly shows that signal transduction, transcription and immune cells response in all cell types are affected by septic plasma. However, modules detected for each cell types appeared to be linked to completely different processes. On the other hand, by analyzing centrality factors, hub genes in these modules were detected, some of them present on the primary list of DEGs. These genes could be considered as critical in the response to sepsis.

Based on all these results, we have provided a table containing all identified hub genes in the PPI or coexpression networks, their correlation to sepsis, and the main transcription factors (Table 1). Additionally, through vigorous search in the available databases such as NCBI, we have introduced the genes specifically involved in immune or inflammatory responses to sepsis. Resultantly, SOCS1, CDKN1A, GNAQ, IL1B, JUN, MAP2K1, MAPK14, SMAD3, SRC, CXCR5, and STAT1 were extracted as hub genes in the PPI networks, many of them known to be involved in modulation of immune system. For example, it has been established that SOCS1 suppresses TLRs and cytokine receptors and regulates metabolic reprogramming in DCs to protect different organs from damages caused by dysregulated inflammatory response during sepsis [38]. Furthermore, ADORA3 and CD83 were present as the hubs in all cell types, verifying the role of ADORA3 in pro-and anti-inflammatory responses [39]. On the other hand, CD83 is a marker for maturation of DCs and its expression level is shown to be lower in septic patients compared to the healthy controls [40].

**Table 1:** DEGs involved in inflammatory/immune response. This table was provided by checking the differentially expressed hub genes in all networks for being involved in inflammatory/immune response. In addition, we checked to datasets to detect if hub genes in this study are also deregulated in others. Abbreviations: PPI: Protein-Protein Interaction; WGCNA: Weighted Gene Co-expression Network Analysis; DCs: Dendritic Cells; NTs: Neutrophils; PBMCs: Peripheral Blood Mononuclear Cells.

Cell Type	Gene symbol	Log2FC (This study)	WGCNA (Hubs)	PPI network (Module No.)	PPI network (Hubs)	TFs from GRN	GSE26378 (Log2FC)	GSE54514 (Log2FC)	PMCID/PMID
**DCs**	ADORA3*	1.30	✓	-	-	NR1H3	1.40	-	10660615
	ATF3	-0.97	-	✓	-	ATF3	-	-	PMC2783469
	CD163	1.37	✓	-	-	-	2.39	-	PMC3638564
	CD163L1	1.06	✓	-	-	-	-	-	25877931
	**CD83**	-0.99	✓	-	-	RUNX2	-	-	23787022
	FKBP5	1.42	✓	-	-	NR1H3, RUNX2	2.26	-	PMC4156438
	SATB1	-0.71	✓	-	-	RUNX2	-1.01	-	26667169
	SOCS1	0.77	-	✓ (M2)	✓	-	-	0.70	PMC5499360
	TIMP3	-1.42	✓	✓	-	-	-	-	16393953
	TNFRSF9	-1.02	✓	✓	-	KLF4, ATF3	-	-	7678621
**NTs**	**ADORA3**	1.09	✓	-	-	-	1.40	-	10660615
	CCL3	-1.27	✓	✓	-	-	-	-	PMC98771
	CD300LF	-0.84	✓	-	-	-	1.17	-	PMC3596959
	**CD83**	-0.85	✓	-	-	-	-	-	23787022
	CDKN1A	-0.80	-	✓ (8)	✓	SMAD3	-	-	12972156
	CEBPD	1.32	✓	✓	-	BCOR	1.54	-	9792624
	CLEC4D	1.47	✓	-	-	-	2.96	-	PMC4589735
	CRISPLD2	0.66	✓	-	-	-	-	-	PMC5027350
	ELK1	-0.9	✓	✓	-	-	-	-	PMC5519339
	FKBP5	2.81	✓	✓ (8)	-	-	2.26	-	PMC4156438
	GNAQ	1.07	-	✓	✓	-	1.31	-	PMC4068741
	HVCN1	-0.61	✓	-	-	-	-0.93	-	PMC4172129
	IL1B	-1.39	-	✓ (4)	✓	-	1.15	-	PMC3083294
	JUN	-0.66	-	✓	✓	-	-	-	21339212
	MAP2K1	0.99	-	✓	✓	-	-	-	10679258
	MAPK14	0.86	-	✓	✓	BCOR	2.38	-0.83	PMC4264013
	NLRP6	1.27	✓	✓	-	-	-	-	PMC3810296
	RNASE6	1.17	✓	-	-	-	-0.79	-	PMC4281292
	SH2B3	-0.92	✓	-	-	-	-	-	PMC4393357
	SMAD3	-0.68	-	✓	✓	SMAD3	-0.91	-	PMC3634534
	SRC	-1.67	-	✓	✓	-	-	-	PMC3893689
	TOB1	1.27	✓	✓	-	SMAD3	-	-	PMC4111268
**PBMCs**	**ADORA3**	1.75	✓	-	-	GATA2	1.40	-	10660615
	ATF3	-0.77	-	✓	-	ATF3	-	-	PMC2783469
	CCL3	-0.68	✓	✓	-	ATF3	-	-	PMC98771
	CD40	-0.74	✓	-	-	-	-	-	PMC4831216
	**CD83**	-0.72	✓	-	-	-	-	-	23787022
	CD163	2.38	✓	-	-	-	2.39	-	PMC3638564
	CEBPD	0.84	✓	✓ (4)	-	-	1.54	-	9792624
	CXCL11	-1.82	✓	✓ (2)	-	-	-	-	PMC4348563
	CXCR5	-0.77	-	✓ (2)	✓	-	-	-	PMC2759473
	FASLG	-0.67	✓	✓ (6)	-	-	-	-	PMC3519983
	FFAR2	-1.24	✓	✓ (3)	-	-	1.70	-0.82	PMC4734206
	GATA2	-0.85	-	-	-	GATA2	-0.62	-	PMC4409096
	GBP1	-1.03	✓	✓ (1)		ATF3	-	-	PMC5307439
	GBP4	-1.05	✓	✓ (1)		-	-0.83	-	PMC3570361
	GBP5	-1.07	✓	✓ (1)	-	ATF3	-	-	26996307
	IDO1	-1.31	✓	-	-	ATF3	-	-	PMC4586474
	IFI35	-0.62	✓	✓ (1)	-	-	0.95	-	PMC5643540
	IFIH1	-0.62	✓	-	-	-	-	-	PMC3262963
	ISG15	-1.00	✓	✓ (1)	-	-	-	2.5	PMC522249
	LTB	-0.84	✓	-	-	-	-0.81	-0.88	12474234
	OAS2	-0.97	✓	✓ (1)	-	-	-	2.17	PMC4351405
	RIPK2	-0.61	✓	✓ (6)	-	-	-	-	PMC4579271
	SAMD9L	-0.76	✓	-	-	-	0.78	1.14	PMC5399482
	SIGLEC9	0.69	✓	-	-	-	1.56	-	10801862
	SLA	0.61	✓	-	-	-	1.24	-	PMC4137477
	SLAMF7	-1.09	✓	-	-	-	-	-	23695528
	STAT1	-0.60	-	✓ (1)	✓	-	-	0.89	PMC3670275
	TLR7	-1.19	✓	-	-	-	-0.63	-	15585605
	TNFRSF9	-0.94	✓	✓	-	ATF3	-	-	PMC3104000

* Bold symbols shows repetitive genes in all cell types.

Independent verification of these results carried out by analyzing two other transcriptome data [17]. Similar expression patterns were detected for ADORA3, CD163, FKBP5, SATB1, CEBPD, CLEC4D, GNAQ, HVCN1, MAPK14, SMAD3, GATA2, GBP4, and LTB in the GSE26378 data [17], while opposite expression was observed for CD300LF, IL1B, RNASE6, FFAR2, ISG15, OAS2, SAMD9L, and STAT1. As for the other study, only a few of our genes including SOCS1, MAPK14, FFAR2, ISG15, LTB, OAS2, SAMD9L and STAT1 were deregulated by sepsis. Collectively, FFAR2, MAPK14, LTB, SAMD9L shown dysregulation in all three studies, indicating their roles at both cellular and system-level responses to sepsis. To discuss the specific role of these four genes in the process, we have used previously published data to back up our findings.

FFAR2 is a free fatty acid receptor that is involved in immune response and is expressed in white blood cells. However, it has been linked to the immune response to the nutrient in the pancreas or the intestinal epithelium [41]. MAPK14 is involved in immune response regulation in astrocytes [42], while LTB is expressed in chronic inflammatory conditions and is known as an inducer in the inflammatory response [43]. Our data showed a down-regulation of this gene throughout all studies, which could be due to suppression of immune system to prevent initiation of cytokine storm. SAMD9L activity has been shown in immunodeficiency, MDS and neurological symptoms [44]. However, the expression of this genes was up-regulated in blood samples collected from sepsis patients in contrast to what was observed in PBMCs.

In conclusion, we have performed a comprehensive network-based analysis to identify systems-level responses of main WBCs to sepsis. Our results, clearly showed that sepsis induces deregulation of genes in the immune responses, signal transduction, and apoptosis processes. We have established that sepsis elucidates an immune response not only in NTs but also in PBMCs. Additionally, our study identified a list of differentially expressed hub genes in responses to sepsis, which could be further investigated to find appropriate targets for the treatment of sepsis or for developing markers to determine different stages of the disease.
